# The Effect of Mental Health First Aid Training on Pharmacist and Pharmacy Student Confidence and Knowledge: A Systematic Review and Meta-Analysis

**DOI:** 10.3390/brainsci15080816

**Published:** 2025-07-29

**Authors:** David Frond, Shannon Habba, Brittany Stewart, Kyle J. Burghardt

**Affiliations:** Eugene Applebaum College of Pharmacy and Health Sciences, Wayne State University, Detroit, MI 48201, USA; davidfrond@wayne.edu (D.F.); smhabba@wayne.edu (S.H.); brittanystewart@wayne.edu (B.S.)

**Keywords:** mental health first aid, systematic review, meta-analysis, pharmacy

## Abstract

**Background/Objectives:** Pharmacists are highly accessible healthcare providers who have frequent, repeated contact with diverse patient populations. They are poised to offer expanded and comprehensive healthcare, including mental health services. One potential barrier to this is a lack of knowledge, confidence, or training in mental health, which may be overcome with a program like Mental Health First Aid (MHFA) training. The aim of this systematic review and meta-analysis is to fill this gap in knowledge by critically evaluating all studies of MHFA training for pharmacists or pharmacy students that report on knowledge, attitudes, or self-efficacy outcomes. **Methods**: A systematic review was performed to identify all relevant studies. Data was extracted and a random-effects meta-analysis was performed for knowledge and attitudes/self-efficacy outcomes, respectively. Subgroup analyses were performed based on survey question type, geographic location, and population studied. **Results**: Overall, MHFA training significantly increased pharmacists’ and pharmacy students’ knowledge (Hedges’ g = 0.228) and combined attitudinal/self-efficacy measures (Hedges’ g = 0.376). Subgroup analyses based on question type, study quality, design, population studied, and location showed similar, significant effects. **Conclusions**: MHFA training appears to have significant effects on pharmacist and pharmacy student knowledge, attitudes, and self-efficacy. Future work should establish the durability of these effects.

## 1. Introduction

Pharmacists often serve as the first point of contact for patients, seeing them twice as often, or more, than primary care physicians [1,2]. This consistent patient interaction places pharmacists in a critical position to recognize and address both physical and mental health concerns, as their role has expanded beyond medication dispensing to encompass more comprehensive care [3]. In 2016, over one billion people globally were estimated to suffer from mental or substance use disorders [4]. The World Health Organization estimates that only 29% of people with psychosis receive mental health services globally, with drastic disparities by income level, as 70% receive care in high-income countries compared to just 12% in low-income settings [5]. For depression, treatment gaps are even wider, as only one third of individuals with major depressive disorder in high-income countries receive formal mental health care [6]. These statistics underscore the urgent need for accessible and well-prepared healthcare professionals to support patients with mental health concerns. Pharmacists, given their accessibility and trust within communities, are well-suited to help meet this need, particularly as public stigma declines and help-seeking behavior increases.

Despite their front-line role, many pharmacists report feeling underprepared or hesitant when faced with patients experiencing mental health crises. One study revealed that while 58.2% of pharmacists felt that pharmacy school prepared them to manage basic mental health-related pharmaceutical care, 41.4% felt less comfortable addressing mental health issues compared to other conditions like hypertension [7]. This lack of confidence can stem from limited formal education and training in mental health topics or a lack of opportunity to apply concepts in a real-world setting. Enhancing pharmacists’ ability to identify and manage mental health conditions is essential for improving patient outcomes. Building confidence in addressing mental health crises empowers pharmacists to engage more effectively with patients and helps reduce the stigma surrounding mental illness [8]. Without appropriate training, pharmacists may miss early warning signs of mental health crises, contributing to delays in care and worsening patient outcomes.

Several training programs have been developed to enhance pharmacists’ competence in managing mental health, with Mental Health First Aid (MHFA) training standing out as a prominent intervention [9]. MHFA training has been widely implemented across diverse populations, and its effects on the public have been well-documented. Specifically, MHFA training has demonstrated small-to-large improvements in mental health knowledge, recognition of mental disorders, and beliefs about effective treatments [10]. Various health professionals, including nurses and general practitioners, have benefited from MHFA training, showing improvements in confidence and knowledge, and a reduction in stigma [10]. MHFA training is an eight-hour, evidence-based training program designed to help individuals recognize the signs and symptoms of mental health disorders, offer initial support, and refer individuals to appropriate care.

Although MHFA training has been introduced in both pharmacy education and practice, studies focusing specifically on pharmacists and pharmacy students often vary in design, population, and outcome measures. Preliminary findings suggest that MHFA training may improve confidence, knowledge, and willingness to engage with individuals experiencing mental health issues [11]. However, these findings have not yet been synthesized through a formal meta-analysis to determine the intervention’s overall impact. This study seeks to address this gap by conducting a systematic review and meta-analysis to evaluate the effects of MHFA training on pharmacist and pharmacy student confidence, knowledge, and attitudes, providing clearer insight into its potential utility and informing future implementation efforts.

## 2. Materials and Methods

### 2.1. General Approach, Search Strategy, and Inclusion/Exclusion Criteria

This systematic review adheres to the Preferred Reporting Items for Systematic Reviews and Meta-Analyses (PRISMA) guidelines and seeks to answer the following question: What is the impact of MHFA training on the confidence and knowledge of pharmacists and pharmacy students [12]? We hypothesize that MHFA training will significantly improve both confidence and knowledge in these populations, enabling them to better manage mental health issues in patients.

The literature search was conducted using the following databases: PubMed, Web of Science, Embase, and Scopus. Keywords used in the search strategy included “Mental Health First Aid,” “pharmacists,” “mental health training,” “confidence,” and “knowledge.” Searches were conducted in September of 2024, and an updated search was performed in January of 2025. Searches were restricted to English-language articles but not based upon geographic location. The systematic review was registered with PROSPERO (CRD42024596557). Search results were uploaded to Covidence Software for the purposes of screening and extraction. Studies were included if they evaluated the effects of MHFA training on pharmacists or pharmacy students. Acceptable outcomes included knowledge outcomes and survey-based outcomes that assessed self-efficacy or attitudes such as behaviors, confidence, empathy, preparedness, and stigma. Studies were excluded if they did not report relevant outcomes or involved other healthcare professionals without isolating pharmacists’ data. Additionally, studies were excluded if they were reviews. Any study design type was included. Two authors independently screened the titles and abstracts of the identified studies. Full texts of potentially eligible studies were reviewed again by two independent authors, and disagreements were resolved by consensus. Data from the included studies were extracted, including study design, population characteristics, study location, intervention details, and measured outcomes (confidence, knowledge, etc.). Relevant data was extracted from each study and included means, standardized deviations, standard errors, confidence intervals, odds ratios, *p*-values, and other pertinent statistical reporting. If necessary, standard deviations were calculated based on the instructions in the Cochrane Handbook for Systematic Reviews and correlation coefficients were assumed at a conservative 0.5 [13,14].

### 2.2. Data Analysis

Extracted knowledge and survey data outcomes were entered into Comprehensive Meta-Analysis (CMA) software version 4. We determined, a priori, that at least three studies were needed for any meta-analysis to be performed including any subgroup or sensitivity analyses and that cross-sectional studies would be excluded for meta-analyses. Effect sizes in CMA were calculated using Hedges’ g statistic as this measure may provide a more precise estimate for small sample sizes [15]. We decided, a priori, to conduct the meta-analysis starting with two overall analyses based on assessment type (i.e., objective versus subjective assessments). This resulted in an “overall” analysis of the objectively assessed knowledge outcomes and the self-assessed, subjective, survey-based outcomes. As the survey-based outcomes typically assess a variety of attitudes or self-efficacy, we subsequently referred to survey-based outcomes in these broad categories. A random-effects analysis was performed for all combined knowledge and then for all combined survey questions. Since our broad categorization of survey-based questions as attitudinal and self-efficacy is somewhat arbitrary in nature, we performed sub-analyses based on question category (only questions on self-efficacy versus only questions on attitude) and individual question type (e.g., attitudes, behaviors, confidence, etc.) to better understand if the overall meta-analyses were reflective of individual survey-based question types. Furthermore, to understand whether the overall meta-analyses were robust to various confounding factors, we performed subgroup and sensitivity analyses that utilized study location, study design, study quality, and population type as grouping factors. All meta-analyses were tested by a two-sided test with statistical significance set at *p* < 0.05. Heterogeneity was tested using the Q value and I^2^ statistics with significant heterogeneity defined as a Q statistic *p* < 0.05 and/or I^2^ greater than 50%. Publication bias was assessed using funnel plots, trim-and-fill analyses, Egger’s test, and the fail-safe N.

For included studies that were cross-sectional in nature, a qualitative summary was performed that included the outcomes of each study. These were not included in the meta-analysis because of the reduced ability to establish causality and potential confounding effects on the meta-analysis.

### 2.3. Study Quality

Given that this systematic review included studies of varied designs, we utilized the NIH Study Quality evaluation templates for each given study design to evaluate the risk of bias and study quality [16]. We categorized studies of high quality as having 75% or greater “yes” answers to the quality criteria, while moderate-quality studies had 50–74% and low-quality ones had less than 50% adhering to quality criteria. Publication bias was evaluated statistically as described above.

## 3. Results

### 3.1. Overview of Included Studies

A total of 3584 references were identified for screening. After duplicate removal, 1667 studies were screened against the title and abstract, with 1611 excluded. Full-text eligibility was assessed for 52 studies, with 36 studies excluded for various reasons, including 11 studies not being relevant to the review question. A total of 16 studies were included in the review (PRISMA Flow Diagram in Supplementary Figure S1). Of these included studies, two were randomized controlled trials with a pre–post design, one was a non-randomized, controlled trial with a pre–post design, eight had a pre–post design without a control group, two had a post-only design with a control group, and three had a post-only design without a control group [17,18,19,20,21,22,23,24,25,26,27,28,29,30,31]. Table 1 provides a description of the studies included.

### 3.2. The Effect of MHFA Training on Knowledge in Pharmacy Populations

**Figure 1:**
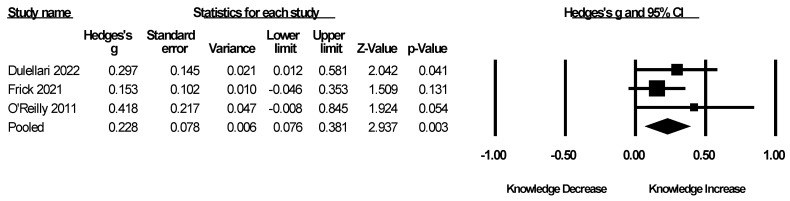
Forest plot of knowledge meta-analysis [19,21,26].

Knowledge was assessed in three studies (Table 2) [19,21,26]. The meta-analysis demonstrated an overall significant effect of MHFA training on knowledge (g = 0.228, CI [0.076, 0.381], *p* = 0.003, Figure 1). The heterogeneity was determined to be low and statistically non-significant (Q = 1.526, I^2^ = 0%). Trim-and-fill analyses adjusted the overall effect from 0.228 to 0.153; however, little asymmetry was found (Egger’s intercept = 2.42, *p* = 0.126) and the fail-safe N was 5.

### 3.3. The Effect of MHFA Training on Attitudinal and Self-Efficacy Measures in Pharmacy Populations

**Figure 2:**
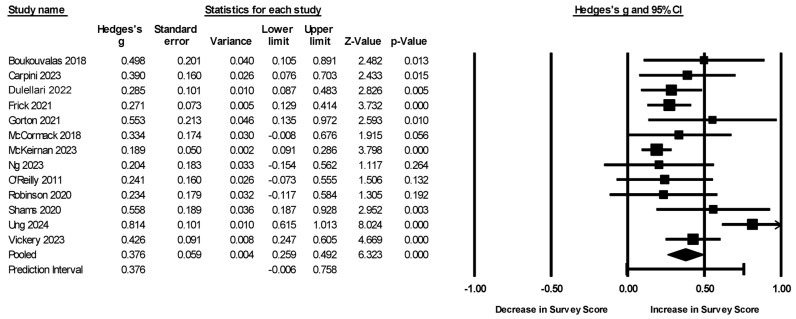
Forest plot of attitudinal and self-efficacy meta-analysis. The prediction interval is an estimate of the range in which a new study may fall accounting for heterogeneity in the observed studies. Therefore, assuming the g units are normally distributed, we would estimate that the true effect size in 95% of all comparable populations would fall between −0.006 and 0.758 [17,18,19,21,22,23,24,25,26,27,28,29,32].

All included studies assessed attitudinal and/or self-efficacy outcomes through surveys. The combined (any survey measure) meta-analysis showed a significant improvement in these measures (g = 0.376, CI [0.259, 0.492], *p* < 0.001, Figure 2). The heterogeneity of this combined analysis was high and significant (Q = 37.23, *p* < 0.001, and I^2^ = 68%), suggesting that the true effect size was not uniform across the studies. Trim-and-fill analyses had no effect on the estimate, and little asymmetry was found (Egger’s intercept = 1.47, *p* = 0.177). The fail-safe N was 391.

When performing meta-analyses based on the categories of attitudes and self-efficacy, respectively, the results remained statistically significant (attitudes: g = 0.298, CI [0.211, 0.385], *p* < 0.001; and self-efficacy: g = 0.561, CI [0.230, 0.891], *p* = 0.001). The heterogeneity for the attitude analysis was low and non-significant (Q = 15.39, *p* < 0.221, and I^2^ = 22%), while the heterogeneity for the self-efficacy analysis was high and significant (Q = 42.45, *p* < 0.001, and I^2^ = 91%). For the attitude analysis, trim and fill reduced the effect from 0.298 to 0.249, with identified asymmetry (Egger’s intercept = 2.03, *p* = 0.022) and a fail-safe N of 196. For the self-efficacy analysis, trim and fill had no effect on the estimate, with little asymmetry identified (Egger’s intercept = 7.40, *p* = 0.1). The fail-safe N was 116.

### 3.4. Subgroup, Moderator, and Sensitivity Analyses for Survey Measures

Further analyses were performed based on survey question type and other potential moderators (Table 3). These analyses were performed if there were at least three studies available; therefore, these analyses were not performed for the knowledge meta-analysis.

First, we looked at the survey question subtypes. Overall, all question subtypes were significant. A high degree of heterogeneity was observed for questions on confidence and a moderate degree of heterogeneity for questions on stigma. To further assess possible factors contributing to this high heterogeneity in the confidence analysis, we performed further sensitivity analyses based on study quality, study design, and removal of potential outliers. When analyzing confidence in studies of lower quality (five studies with less than 75% concordance with quality criteria), the results remained significant (g = 0.339, CI [0.209, 0.468], *p* < 0.001). Heterogeneity was considered non-significant and low (Q = 5.654, I^2^ = 29%). When analysis was restricted to studies assessing confidence that included a control group (N = 3), confidence remained significantly increased but slightly lower than the overall confidence meta-analysis (g = 0.450, CI [0.246, 0.655], *p* < 0.001). Heterogeneity was considered non-significant and low (Q = 2.019, I^2^ = 1%). In contrast, studies assessing confidence without a control group (N = 5), while retaining statistical significance (g = 0.561, CI [0.230, 0.892], *p* < 0.001), showed high and significant heterogeneity (Q = 42.584, I^2^ = 91%, *p* < 0.001). Finally, when performing a leave-one-out analysis, the removal of the Ung 2024 [28] study reduced heterogeneity to 16% (Q = 7.128, *p* = 0.309) with a reduced Hedges’ g of 0.355 (*p* < 0.001). Together, these additional analyses suggest that study design and outliers were contributing to the observed heterogeneity in the confidence analysis.

We also performed an analysis on studies only containing control groups. For the survey results, this included five studies which indicated that MHFA training significantly increased survey scores (g = 0.357, CI [0.199, 0.515], *p* < 0.001). Heterogeneity was considered statistically non-significant and low (Q = 2.603, I^2^ = 0%). Trim-and-fill analyses adjusted the overall effect from 0.357 to 0.325; however, little asymmetry was found (Egger’s intercept = 4.077, *p* = 0.237) and the fail-safe N was 22.

Finally, subgroup analyses by population type (pharmacists versus pharmacy students), study location (United States versus Australia), and study quality (high versus moderate) all yielded significant Hedges’ g effects. Significant heterogeneity was observed for studies in pharmacy students, studies that took place in Australia, and studies of high quality. Similarly to our approach above in the confidence analysis, which showed high heterogeneity, we performed analyses based on study quality and design and outliers. For lower-quality studies in Australia (N = 4), the effect size decreased (g = 0.357, CI [0.180, 0.535], *p* < 0.001) but the heterogeneity was considered non-significant and low (Q = 2.845, I^2^ = 0%). For Australian studies with a control group (N = 4), the effect size decreased (g = 0.325, CI [0.154, 0.495], *p* < 0.001) and the heterogeneity was considered non-significant and low (Q = 1.617, I^2^ = 0%). For Australian studies, an analysis of non-controlled studies could not be performed because there were only two available studies. For lower-quality studies in pharmacy students (N = 8), the effect also decreased (g = 0.247, CI [0.179, 0.315], *p* < 0.001), with low, non-significant heterogeneity (Q = 5.512, I^2^ = 0%). For pharmacy student studies without a control group (N = 7), effect size remained relatively consistent (g = 0.366, CI [0.202, 0.529], *p* < 0.001) but the heterogeneity was high and significant (Q = 32.796, I^2^ = 82%). Performing a leave-one-out analysis demonstrated that removal of the Ung 2024 [28] study from both the pharmacy student and Australian studies reduced the heterogeneity to 10% (Q = 8.889, *p* = 0.352) and 0% (Q = 2.876, *p* = 0.579). Both the pharmacy student and Australian meta-analyses remained significant with Hedges’ g of 0.280 (*p* < 0.001) and 0.365 (*p* <0.001) when removing the Ung 2024 [28] study. This suggests, like the confidence analysis above, that outlier effects and study design are contributing to the observed heterogeneity.

### 3.5. Study Quality Assessment

Study quality was assessed for each study based on the study design using the NIH quality assessment templates. The overall study quality was moderate to high. Quality information is presented in Table 1, and detailed information can be found in the Supplementary Materials.

### 3.6. Narrative Review of Post-Only Studies Without a Control Group

Three studies were cross-sectional in nature, analyzing attitudinal and self-efficacy measures after participants had completed MHFA training [20,30,31]. All assessed behaviors, while two also assessed confidence, one study analyzed preparedness, and one study analyzed reluctance. Witry and colleagues’ study consists of two reports on the same study and was a multi-state analysis of the effect of MHFA training offerings on community pharmacist and pharmacy student outcomes. El Den and colleagues analyzed the effect of an MHFA training offering within a pharmacy program. Within their study, they analyzed behavior through suicide and postnatal depression roleplay evaluations of the Approach, Listen, Give, Encourage (ALGEE) framework [35]. Overall, high degrees of confidence and preparedness, low reluctance, and several behaviors were influenced by MHFA training (Table 4).

## 4. Discussion

This meta-analysis on the effect of MHFA training on pharmacist and pharmacy student knowledge and the combined attitudinal and self-efficacy-based measures showed significant, positive effects in each area with low heterogeneity. The effect size for knowledge was 0.228, while the effect size for the combined attitudinal and self-efficacy measures was 0.376. These effect sizes are considered low to moderate based on traditional Hedges’ g cutoffs. Furthermore, despite the statistical significance of these effects, it is unknown whether these changes have any effects on patient outcomes. For the subgroup analyses of the attitudinal and self-efficacy measures, all analyses remained significant with some subgroup analyses showing high heterogeneity (studies assessing confidence, pre–post without control studies, studies in Australia, studies on pharmacy students, and studies of high quality). The reason for studies with higher concordance with quality criteria displaying higher heterogeneity may be because the quality criteria were specific for the study design, meaning that a study without a control group could still have high criteria concordance even though it is likely to have more bias due to a lack of a control group as compared to a study with a control group. The findings in our analyses do indicate that studies without a control group provided a potential explanation for the high heterogeneity observed in several meta-analyses here (i.e., confidence, location, and population sub-analyses), which emphasizes the need for future studies to have control groups to understand the true effects of MHFA on community pharmacy practice. Our analyses also suggest that outliers, particularly the Ung 2024 [28] study, were a large contributor to the observed heterogeneity. For the studies not included in the meta-analyses (cross-sectional studies without a control group), MHFA training had positive effects on confidence, reluctance, preparedness, and some measures of behavior. Altogether, these analyses demonstrate that MHFA training in pharmacy can have significant positive effects on knowledge, attitudes, and self-efficacy related to mental health.

In addition to pharmacy, MHFA training has also shown positive effects in other healthcare fields. For example, several studies have been performed in nursing student populations with positive effects on confidence, willingness to provide MHFA, stigma, and literacy [36]. Additionally, MHFA also increased nursing knowledge [37]. Positive effects have also been observed on medical student knowledge, intentions, stigma, and confidence [33]. Although it has been found that MHFA training in physician assistant students leads to high satisfaction rates, work has not yet been performed to establish effects on knowledge and attitudes as with pharmacy, medical, and nursing students [38]. These findings demonstrate the effectiveness of MHFA training across the healthcare spectrum, and this, coupled with the positive effect on patient mental health, may give credence to the need to incorporate MHFA training or similar training into all healthcare professional training or to pursue interprofessional efforts in MHFA training [39].

Subgroup analyses were performed, since although other studies have grouped knowledge and survey-based outcomes, as carried out here, this grouping can be considered broad and potentially arbitrary in nature [40]. These subgroup and sensitivity analyses demonstrated a few findings of note. First, MHFA training has significant effects across several attitudinal and self-efficacy domains, suggesting that this relatively simple training has potentially broad effects ranging from increasing confidence and behaviors to being an effective tool for addressing the known issue of mental health stigma within pharmacy and, more broadly, in healthcare [41,42]. Although subgroup analyses were not performed for sub-domains like empathy or preparedness, individual study results do indicate that MHFA training has a significant effect on these areas as well. Given that MHFA training has a significant effect on multiple areas that include both knowledge and attitudes, and given its low cost and structured implementation, it could be used to alter accreditation standards to require mental health training in all pharmacy curricula.

Subgroup findings also demonstrate that MHFA training is significant across the pharmacy profession from students to established, practicing pharmacists, suggesting that such training can be beneficial during training and used as a professional development tool. Similar effects have been demonstrated for nursing students of varying levels of training and work experience [36,43]. This may suggest that MHFA training implementation could happen at any point in a healthcare provider’s career and yield positive effects. However, most studies did assess outcomes immediately or shortly after MHFA training, which does not give any evidence regarding how sustainable these effects are in the long term. Future work could assess the incorporation of MHFA training into continuing education requirements and understand the long-term durability of one-time training versus offering refresher courses over one’s career. Currently, individuals are required to recertify for MHFA training every three years, and it is not known if these recertification trainings result in consistent or variable effects on knowledge and survey-based outcomes.

Subgroup analysis by study location revealed significant differences in the effectiveness of MHFA training between Australia and the United States. In Australian studies, the pooled effect size was notably higher (g = 0.467, 95% CI [0.244, 0.690], *p* < 0.001) with moderate heterogeneity (Q = 15.077, I^2^ = 67%). In contrast, studies from the United States demonstrated a smaller effect size (Hedges’ g = 0.264, 95% CI [0.190, 0.339], *p* < 0.001) and much lower heterogeneity (Q = 5.654, I^2^ = 12%). These results suggest that while MHFA training was effective in both regions, its impact was more variable among Australian populations, which may be attributed to the use of study designs without a control group and an outlier study. Additional contributing factors may include national differences in MHFA training program delivery or cultural attitudes toward mental health. Trim and fill also notably differed, with Australia showing an adjusted g of 0.534 and the U.S. at 0.216. This further emphasizes the role of contextual differences between geographic locations. Given that nearly all the studies included originated from these two countries, there is a clear need for future research to explore MHFA training outcomes in additional geographic regions. A broader international evaluation will be essential to determine the benefit of MHFA training, and adaptations may be needed in more diverse pharmacy settings.

This study has several limitations that should be considered when interpreting the findings and has room for improvement in future studies assessing this topic. First, while the meta-analysis included a large range of studies and study designs, a large portion of these lacked control groups. This lack of control groups limits the ability to infer causality and increases the chance of bias and confounding variables. This was a likely contributor to the high heterogeneity observed in some of the analyses including the overall analysis of survey-based responses, and confidence, location, and population analyses. Further analyses seem to suggest that this heterogeneity may be attributed to study designs that did not utilize a control group, which is further supported by the removal of an outlier study which did not use a control group. Thus, effect interpretation of these studies must be cautious, and future application of these findings, as well as future studies, should rely on controlled studies. Second, all studies relied on self-reported survey data for many key outcomes, which we first grouped into broad categories of knowledge and self-efficacy or attitude. Self-reported data can be highly subjective and may not accurately reflect actual practice and outcomes. We performed many sub-analyses to help us understand whether the significant findings of the overall survey meta-analysis were replicated at the level of each question type. Furthermore, despite the statistical significance of these effects, it is unknown what practical effects such changes in knowledge and survey-based outcomes have on pharmacy student and pharmacist actions, let alone clinical outcomes. Third, due to the data often being self-reported and studies using a varied number of types of assessments, heterogeneity was present, particularly in outcomes such as confidence, with noticeable variability among studies conducted in Australia. This demonstrates the need for the results to be expanded and applied to other countries, accounting for language and cultural differences, for MHFA to have a global effect on community pharmacy practice. The meta-analysis also used a conservative assumption for correlation in some calculations of effect size. A final limitation is that only a small number of studies conducted follow-up assessments to evaluate the long-term effects of MHFA training. Moreover, despite a rigorous search strategy, this review did not include non-English-language publications or unpublished studies. These limitations may be reflected in some of the evidence for asymmetry, potential publication bias, and theoretical effects of unpublished studies on the findings here (small fail-safe values). Such findings indicate that the effect size could be overestimated, thus reducing validity and generalizability, especially outside of community pharmacy populations. Future work must ensure the inclusion of unpublished work when possible, and replication will be critical to this realm of study.

## 5. Conclusions and Future Directions

This meta-analysis suggests that MHFA training has modest effects on community pharmacists’ knowledge and survey-based outcomes like attitudes and self-efficacy. Future research must prioritize more objective and standardized assessments of knowledge and behavioral change to better understand the true meaning of these modest effects both on pharmacist and patient outcomes. Additionally, studies should attempt to understand the durability of these findings using additional follow-up beyond an immediate post-MHFA assessment. Most available studies rely heavily on self-reported surveys, which are subject to bias and may not accurately reflect actual competence in real-world settings. Implementing validated and knowledge-based testing and outcomes along with structured behavioral evaluations would enhance the reliability of outcomes and sustainability. Additionally, many studies did not include control groups, which contributed to the heterogeneity of the analyses and therefore warrants caution in their interpretation, especially as it relates to MHFA’s practical and clinically meaningful effects in community pharmacy. Further structured and controlled longitudinal studies assessing whether behavioral changes are retained and translated into improved patient care are critical for assessing the true impact of MHFA training in the long term within pharmacy practice.

## Figures and Tables

**Table 1:** Overview of included studies.

Study	Study Type	Population	Study Location	Sample Size for MHFA (Control If Applicable)	Outcomes	% Concordance with Quality Criteria
Boukouvalas 2018 [17]	Pre–post with control	Pharmacy students	AUS	40 (146)	Attitudes, confidence	57
Carpini 2023 [18]	Post with control	Pharmacists	AUS	90 (71)	Barriers, behaviors, confidence	75
Dulellari 2022 [19]	Pre–post without control	Pharmacy students	USA	53	Confidence, knowledge, perceptions	67
El-Den 2018 [20]	Post without control	Pharmacy students	AUS	143	Behaviors, confidence	57
Frick 2021 [21]	Pre–post without control	Pharmacy students	USA	135	Attitudes, confidence, empathy, knowledge, social distancing	67
Gorton 2021 [22]	Post with control	Pharmacy students	UK	26 (205)	Preparedness, stigma	67
McCormack 2018 [23]	Pre–post without control	Pharmacy students	USA	34	Attitudes, social distancing	58
McKeirnan 2023 [24]	Pre–post without control	Pharmacy students	USA	212	Comfort, confidence, stigma	67
Ng 2023 [25]	Pre–post with control	Pharmacists	AUS	59 (81)	Attitudes, barriers, behaviors, confidence, social distancing	71
O’Reilly 2011 [26]	Pre–post with control	Pharmacy students	AUS	53 (170)	Beliefs, behaviors, knowledge, social distancing	50
Robinson 2020 [32]	Pre–post without control	Pharmacy students	USA	40	Attitudes, behaviors, social distancing	67
Shams 2020 [27]	Pre–post without control	Pharmacists	AUS	32	Attitudes, behaviors	67
Ung 2024 [28]	Pre–post without control	Pharmacy students	AUS	148	Confidence, social distancing	75
Vickery 2023 [29]	Pre–post without control	Pharmacy students	USA	69	Benefits, confidence	75
Witry 2020a [30]	Post without control	Pharmacists and pharmacy students	USA	96	Behaviors, preparedness	50
Witry 2020b [31]	Post without control	Pharmacists and pharmacy students	USA	98	Behaviors, confidence, reluctance	50

**Table 2:** Overview of studies assessing knowledge.

Study	Number of Questions	Answer Type	Topics	Previously Used?
Dulellari 2022 [19]	16	Agree or Disagree	Depression, anxiety, psychosis, and substance misuse.	Yes [33]
Frick 2021 [21]	10	True or False	Medications, identifying various types of mental illnesses, and recognizing misconceptions related to mental illness.	Yes [34]
O’Reilly 2011 [26]	1 (open-ended)	Correctly Identified Disorder	Identification of depression and schizophrenia disorders in vignettes.	No

**Table 3:** Results for subgroup analyses.

Subgroup Variable	Number of Studies	Hedges’ G [95% CI]	Effect Size *p*-Value	Q/I^2^	Q Statistic *p*-Value	Trim-and-Fill Hedges’ G	Egger’s Intercept	Fail-Safe N
Subgroup Analysis by Question Type								
Attitude	7	0.241 [0.127, 0.355]	<0.001	3.841/0%	0.698	0.241	1.476	26
Behavior	5	0.280 [0.123, 0.438]	<0.001	4.457/10%	0.348	0.321	7.309	14
Confidence	8	0.521 [0.288, 0.753]	<0.001	44.787/84%	<0.001	0.593	4.147	26
Social Distancing Scale	6	0.302 [0.188, 0.415]	<0.001	4.969/0%	0.420	0.326	−0.397	34
Stigma	3	0.226 [0.027, 0.424]	0.026	3.948/49%	0.139	0.201	0.817	6
Subgroup Analysis by Study Design								
Post with Control	3	0.376 [0.171, 0.582]	<0.001	1.340/0%	0.512	0.320	2.690	8
Pre–Post without Control	8	0.383 [0.228, 0.537]	<0.001	34.434/80%	<0.001	0.415	2.320	215
Pre–Post with Control	3	0.297 [0.095, 0.500]	0.004	1.385/0%	0.500	0.297	5.390	4
Subgroup Analysis by Location								
Australia	6	0.467 [0.244, 0.690]	<0.001	15.077/67%	0.010	0.534	−4.910	84
USA	6	0.264 [0.190, 0.339]	<0.001	5.654/12%	0.341	0.216	1.246	81
Subgroup Analysis by Population Type								
Pharmacy Students	10	0.376 [0.239, 0.512]	<0.001	35.073/74%	<0.001	0.417	1.732	271
Pharmacists	3	0.381 [0.182, 0.580]	<0.001	1.817/0%	0.403	0.309	1.473	9
Subgroup Analysis by Study Quality								
Moderate	10	0.255 [0.190, 0.321]	<0.001	8.212/0%	0.513	0.225	1.306	143
High	3	0.553 [0.272, 0.834]	<0.001	9.532/79%	0.009	0.568	−1.661	57

**Table 4:** Outcomes for cross-sectional studies.

Study	Outcomes
El-Den 2018 [20]	For all confidence questions, at least 84% of students agreed or strongly agreed with each confidence prompt. Five confidence prompts showed 95% of students agreeing or strongly agreeing. For the analysis of behaviors, at least 80% of students performed the correct behavior for 7 of the 10 assessed suicide behaviors and 7 of the 11 assessed postnatal depression behaviors.
Witry 2020a [30]	For preparedness assessments, all prompts had at least 74% of participants agreeing or strongly agreeing that they were prepared to perform the behavior after MHFA training. Seven of thirteen behaviors had at least 90% agreeing or strongly agreeing for preparedness. Regarding behaviors, 80% or more participants reported performing two of seven behaviors.
Witry 2020b [31]	For reluctance measures, at least 74% disagreed or strongly disagreed that they were reluctant to perform an action for 3 of the 6 items assessed. For confidence, all 7 measures showed at least 82% agreeing or strongly agreeing that they were confident. For self-reported behaviors, at least 70% of participants responded yes for performing 3 of 9 behaviors.

## Data Availability

These data were extracted from published studies. Extracted data is available upon request from the authors.

## Supplementary Materials

The following supporting information can be downloaded at https://www.mdpi.com/article/10.3390/brainsci15080816/s1, Figure S1: PRISMA flow diagram; Table S1: Quality review for observational and cohort Studies; Table S2: Quality review for case control studies; Table S3: Quality review for controlled intervention studies; Table S4: Quality review for pre–post studies without a control group.

## Abbreviations

The following abbreviations are used in this manuscript:

CI	Confidence Interval
MHFA	Mental Health First Aid
CMA	Comprehensive Meta-Analysis
PRISMA	Preferred Reporting Items for Systematic Reviews and Meta-Analyses
